# Sperm competition in yellow dung flies: No consistent effect of sperm size

**DOI:** 10.1111/jeb.14073

**Published:** 2022-08-16

**Authors:** Ane T. Laugen, David J. Hosken, Klaus Reinhold, Gioia A. Schwarzenbach, Paquita E. A. Hoeck, Luc F. Bussière, Wolf U. Blanckenhorn, Stefan Lüpold

**Affiliations:** ^1^ Department of Evolutionary Biology and Environmental Studies University of Zurich‐Irchel Zurich Switzerland; ^2^ Centre for Coastal Research, Department of Natural Sciences University of Agder Kristiansand Norway; ^3^ Centre for Ecology and Conservation University of Exeter in Cornwall Penryn UK; ^4^ Evolutionsbiologie Universität Bielefeld Bielefeld Germany; ^5^ Biology and Environmental Sciences University of Gothenburg and Gothenburg Global Biodiversity Centre Gothenburg Sweden

**Keywords:** body size, cryptic female choice, fertilization success, P_2_, sexual selection, sperm competition, sperm length, temperature

## Abstract

The male competition for fertilization that results from female multiple mating promotes the evolution of increased sperm numbers and can impact sperm morphology, with theory predicting that longer sperm can at times be advantageous during sperm competition. If so, males with longer sperm should sire more offspring than competitors with shorter sperm. Few studies have directly tested this prediction, and findings are inconsistent. Here we assessed whether longer sperm provide a competitive advantage in the yellow dung fly (*Scathophaga stercoraria*; Diptera: Scathophagidae). Initially, we let brothers with different temperature‐mediated mean sperm lengths compete – thus minimizing confounding effects of genetic background – and found no clear advantage of longer sperm. We then used flies from lines subjected to bidirectional selection on phenoloxidase activity that had shown correlated evolutionary responses in sperm and female spermathecal duct lengths. This experiment also yielded no main effect of sperm size on siring success. Instead, there was a trend for a shorter‐sperm advantage, but only when competing in females with longer spermathecal ducts. Our data corroborated many previously reported findings (last‐male precedence, effects of copula duration and body size), suggesting our failure to find sperm size effects is not inherently due to our experimental protocols. We conclude that longer sperm are not competitively superior in yellow dung flies under most circumstances, and that, consistent with previous work, in this species competitive fertilization success is primarily determined by the relative numbers of sperm competing.

## Graphical abstract

Males vary in sperm size and shape within and between species. Here, we tested whether longer sperm provide a competitive advantage in the yellow dung fly (Scathophaga stercoraria), based on two different experimental manipulations of sperm length. We found no clear competitive advantage of longer sperm; rather, the effect of sperm length on competitive fertilization success varied with the size of females or their sperm‐storage organs. More than by variation in sperm length, competitive fertilization success was determined by the relative numbers of sperm competing.

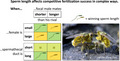

## 1 INTRODUCTION

Most female animals mate with more than one male during a typical reproductive bout, and this can lead to sperm competition (Parker, 1970). A near‐universal response to this post‐mating male–male competition is increased sperm production (and transfer to females) to gain a numerical advantage over competing ejaculates (Birkhead & Møller, 1998; Lüpold, de Boer, et al., 2020; Parker, 1982; Parker & Pizzari, 2010; Simmons & Fitzpatrick, 2012). There can be additional selection on other ejaculate characters, such as the composition of the seminal fluid or the form and function of sperm (Fitzpatrick & Lüpold, 2014; Parker, 1993; Pizzari & Parker, 2009; Snook, 2005; Wigby et al., 2020).

Sperm morphology, particularly sperm size, is one prominent and intensely studied aspect likely to affect competitive fertilization (reviewed in Fitzpatrick & Lüpold, 2014; Lüpold & Pitnick, 2018; Pitnick, Hosken, & Birkhead, 2009). Studies testing for associations between post‐mating sexual selection and sperm length are primarily based on interspecific comparisons (Lüpold, de Boer, et al., 2020; Minder et al., 2005; Simmons & Fitzpatrick, 2012) or experimental evolution with and without sperm competition (polyandry vs. monogamy: e.g. Firman & Simmons, 2010; Hosken et al., 2001; LaMunyon & Ward, 2002; Pitnick et al., 2001; ). Parker's (1993) original model predicted sperm length to evolve only under very special conditions. Perhaps for this reason, direct evidence for sperm size effects on ejaculate competitiveness within species remains rather limited, with mixed results. Although some studies have shown that larger or longer sperm are more competitive (Bennison et al., 2015; LaMunyon & Ward, 1998; Lüpold et al., 2012; Miller & Pitnick, 2002; Oppliger et al., 2003; Radwan, 1996), others report a competitive advantage for shorter sperm (Gage & Morrow, 2003; García‐González & Simmons, 2007) or find no effect of sperm size (Boschetto et al., 2011; Dziminski et al., 2009; Laskemoen et al., 2010; Morrow & Gage, 2001; Simmons et al., 2003).

Although existing evidence for post‐mating sexual selection on sperm size seems rather inconsistent, theoretical models predict that sperm competition can favour either smaller or larger sperm, depending on how sperm compete (Immler et al., 2011; Parker et al., 2010). For example, if sperm competition follows the raffle principle, where more sperm increase the likelihood of siring success, selection should favour smaller sperm to maximize sperm number (assuming a size−number trade‐off; Parker, 1982), as found, for example, in crickets (Gage & Morrow, 2003) or dung beetles (García‐González & Simmons, 2007). However, longer sperm can also provide competitive advantages, for example if they swim faster (interspecific evidence: Gomendio & Roldan, 2008; Fitzpatrick et al., 2009; Lüpold et al., 2009; intraspecific evidence: Malo et al., 2006; Mossman et al., 2009; Fitzpatrick et al., 2010) or are better at displacing rival sperm from female sperm‐storage organs (Lüpold et al., 2012; Miller & Pitnick, 2002). In fact, sperm displacement is a widespread sperm competition mechanism in insects (Ridley, 1989) that can underpin the evolution of exceptionally long sperm (Immler et al., 2011; Lüpold & Pitnick, 2018; Parker et al., 2010), with frequent coevolution occurring with elements of the female reproductive tract (Dybas & Dybas, 1981; Higginson et al., 2012; Lüpold et al., 2016; Minder et al., 2005; Morrow & Gage, 2000; Thüler et al., 2011).

If longer sperm confer an advantage in competitive fertilization, an obvious, direct prediction is that males with longer sperm should sire more offspring than sperm competitors producing smaller sperm. We tested this prediction in the yellow dung fly *Scathophaga stercoraria*, the classic model species for studies of sperm competition and sexual selection for over 50 years (Simmons et al., 2020). In this species, sperm vary considerably in length due to both environmental and genetic effects (Blanckenhorn & Hellriegel, 2002; Hellriegel & Blanckenhorn, 2002; Ward, 2000; Ward & Hauschteck‐Jungen, 1993). Previous work has documented an influence of sperm length on sperm storage by yellow dung fly females, but did not investigate subsequent paternity (Otronen et al., 1997). Moreover, sperm length also showed no short‐term micro‐evolutionary response to experimental manipulation of sperm competition risk (Hosken et al., 2001), so as yet there is no evidence for greater competitiveness of longer sperm in this species.

Combining two independent experiments in the yellow dung fly, we here directly tested whether sperm length affects paternity during sperm competition. Initially, we relied on environmentally mediated sperm length variation while simultaneously minimizing potential confounding effects of genetic background by competing brothers of different sperm lengths with one another. We then capitalized on a correlated genetic response of sperm length to artificial selection on phenoloxidase (PO) activity (Schwarzenbach, 2006; Schwarzenbach & Ward, 2006, 2007). For unknown reasons, but perhaps because of trade‐offs (see Hosken, 2001), selection for low PO activity resulted in males with longer sperm and females with longer spermathecal ducts in each of three replicate selection lines. By contrast, selection for high PO activity resulted in no changes to either trait. In both experiments, we applied molecular paternity analyses (Bussière et al., 2010; Demont et al., 2011, 2012, 2021; Garner et al., 2000).

## 2 MATERIALS AND METHODS

All flies in the experiment using environmentally mediated sperm length variation among competing brothers stemmed from laboratory cultures held and reared at standard conditions for 2–3 generations (18°C, 60% humidity, 13 h photoperiod). To ascertain brothers with different sperm lengths, we split the clutch of any mother to rear one half at 15°C and the other at 23°C, as temperature had previously been shown to systematically affect sperm length in yellow dung flies (Blanckenhorn & Hellriegel, 2002). For our matings performed at room temperature (20–22°C), we selected one random male per family emerging from each temperature treatment to compete for fertilization in a random female from another random family. Experimental adults were held for ca. 2 weeks after emergence under ad libitum food conditions until they reached sexual maturity.

We used two slightly different experimental designs: paired and unpaired. In the paired design, we allowed the same pair of brothers to first copulate in a particular (random) order with a non‐related female, and thereafter in reverse order with one of her sisters. In the unpaired design, pairs of brothers were randomly assigned to a copulating sequence (long‐spermed males first or last), but each pair competed only once. Our tests were blind because we did not know sperm length until after the experiment. For the mating trials we placed a female in a small glass vial, added one of the males, and then recorded copulation duration to the nearest minute before replacing the male. In the paired data set, we gave the males at least 30 min to recover before pairing them with the second female in reverse mating order.

In the tests using selection lines, flies stemmed from populations that had been subjected to bidirectional artificial selection on PO levels. Whereas all three replicate lines selected for high PO concentration generated flies with short sperm (in males) and short spermathecal ducts (in females) as a correlated response, selection for low PO levels produced male flies with long sperm and female flies with long spermathecal ducts (Schwarzenbach, 2006; Schwarzenbach & Ward, 2006, 2007). After 13 generations of selection, we paired randomly picked flies among the three replicate lines within each PO selection regime to offset any potential inbreeding effects within lines. From these crosses, we derived three new crossed experimental lines (i.e. ‘short‐sperm’ lines from the three high PO crosses, and ‘long‐sperm’ lines from the three low PO crosses) to stage competitive matings between them. In brief, we allowed virgin females of either selection regime to sequentially mate with two sexually naïve males, one from each regime, half with short‐sperm males mating first, and half with long sperm males first. Within each mating trio, the male and female of the same selection regime came from separate line crosses, in all possible combinations between regimes, lines and sexes. As in the tests competing brothers, we combined each virgin female with their first male in a glass vial and supplied the second male after successful initial copulation.

In both experiments, we allowed each double‐mated female to oviposit her first clutch of eggs into a smear of fresh dung on a filter paper (typically within 30 min of the second copulation), which we then transferred into a plastic container with abundant dung for larval development at 18°C. We measured the length of one hind tibia of all individuals as a measure of body size (Simmons & Ward, 1991) and froze all parents and emerging offspring at −80°C for later measurement of internal morphology (described below; experiment 1 only), DNA extraction and genotyping. We used as many microsatellite loci as needed to unequivocally assign paternity to one or the other male following well established protocols and using Applied Biosystems *GeneMapper* software (Bussière et al., 2010; Demont et al., 2011, 2012, 2021). We typically genotyped a random subset of 16–20 offspring from a female's first clutch (typically comprising 30–70 eggs). Since approximately 15% of all families produced only partial (i.e. small) clutches, we included all families with at least eight offspring.

For each competing brother in the first experiment, we removed both testes in insect Ringer solution, released the sperm from the proximal third of the testis (relative to the ejaculatory duct) into a drop of solution on a microscope slide, and measured the total length (head plus tail) of 20 sperm using ImageJ to compute his mean sperm length (Hellriegel & Blanckenhorn, 2002; Ward & Hauschteck‐Jungen, 1993). We further measured the lengths of all three spermathecal ducts per female and the area (length and width) of their corresponding spermathecae. We also took the same measurements for a random subsample of 15 individuals per replicate line in the second experiment. Although these were not the specific individuals used in our sperm competition experiment, their sperm and spermathecal ducts had diverged to disparate length after the 13 generations of selection. Specifically, sperm lengths averaged 216.8 ± 0.95 and 212.3 ± 0.62 μm in the low and high PO selection regimes, the corresponding spermathecal duct lengths being 708 ± 6.8 and 679 ± 18.7 μm, respectively (means ± SE of three replicate lines, *N* = 45; Schwarzenbach, 2006).

### 2.1 Statistical analyses

Although biologically realistic (Simmons, 2001; Simmons & Siva‐Jothy, 1998), second‐male offspring proportions (P_2_) of 0 or 1 may result from one of the two matings being unsuccessful, complete sperm displacement (for P_2_ = 1) or from total male infertility. We excluded five brother pairs in the paired data set of the first experiment for which the same competitor achieved no paternity whatsoever, thus potentially indicating infertility. For the remaining trials, we conducted our analyses both including and excluding cases of P_2_ = 0 or 1, as it was not possible to ascertain successful sperm transfer.

For all analyses of relative paternity shares, we performed generalized linear mixed models (GLMMs) with a binomial error distribution and logit link function in R v.4.1.2 (R Core Team,  2021) using the *mixed* function in the *afex* package (Singmann et al., 2021), which calls the *glmer* function (*lme4* package; Bates et al., 2015) to calculate coefficients and *PBmodcomp* (*pbkrtest* package; Halekoh & Højsgaard, 2014) for parametric bootstrapping of *p*‐values across *N* = 1000 simulations. For the first experiment, the final GLMM with binomial error distribution and logit link function included the proportion of offspring sired by the second male as a paired dependent variable [cbind(second, first)], data set (paired or unpaired assay), female body size and the relative differences [(second − first) / (first + second)] in body size, copula duration and sperm length between the competitors. We initially also included the two‐ and three‐way interactions between female body size and the relative differences in copula durations and sperm lengths, but subsequently dropped non‐significant interactions from our final models. We considered these interactions because female size predicts the size of the sperm‐storage organs and thus their storage capacity (Parker et al., 1999; Schwarzenbach, 2006; Thüler et al., 2011) and so potentially the degree of sperm displacement (Lüpold, Reil, et al., 2020), and it might itself result in sperm allocation by males due to size‐dependent fecundity (Kelly & Jennions, 2011; Wedell et al., 2002). Sperm transfer (and ultimately paternity) increases with copula duration (Demont et al., 2021; Parker & Simmons, 1994; Simmons et al., 1999), which could interact with female size (above) or with relative sperm lengths, capturing potential trade‐offs between sperm size and number. Finally, the interaction of relative sperm lengths and female size could provide information on differential sperm competitiveness in response to female sperm‐storage structures (Miller & Pitnick, 2002). To account for the non‐independence of data for competing brothers tested in both mating orders in the paired assay, we included male family identity (brother pair) as a random effect, plus an observation‐level random effect served to mitigate overdispersion.

The second experiment was based on *N* = 126 double matings, though missing data reduced this data set to *N* = 117 for our analyses. Here, we analogously performed our final GLMM on the proportion of offspring sired by the second male (P_2_; paired variable) with female and last‐male selection regimes as fixed factors (including their interaction), female body size, the relative differences in body size and copula duration between the two males as fixed effects, and male × female replicate line combinations (*N* = 24) and experimental block as random effects. Again, an observation‐level random effect addressed overdispersion. Note that sperm and spermathecal duct lengths had been pre‐determined in other flies from the selection lines and found to be distinct; hence, we did not additionally include these variables in our analysis because they were subsumed in the fixed effect of selection regime.

## 3 RESULTS

### 3.1 Competing brothers with varying temperature‐mediated sperm length

Across the *N* = 53 unique brother–brother comparisons, including 24 in the unpaired and 29 in the paired assays, mean sperm length of long‐spermed males was 214.3 [95%CI = 213.0–215.5] μm compared to 210.3 [209.1–211.5] μm of their short‐spermed brothers (paired *t*‐test: *t*_52_ = 9.28, *p* < 0.0001), with the longer sperm exceeding the shorter by 0.02–5.14% within comparisons.

**FIGURE 1:**
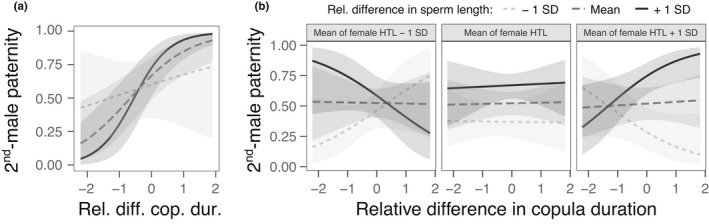
(a) The predicted proportion of offspring sired by the second male P_2_ (with 95% confidence bands) increases with the relative difference in copula durations and in sperm lengths between the competing brothers (first experiment), (b) whereby female body size (HTL: Hind‐tibia length) incurs additional complex effects. To depict interactions among the continuous variables, sperm length and female body size were partitioned into three bins representing the mean, −1 SD smaller, and +1 SD larger than the mean values. The two‐way interaction in panel a is based on the full data set (*N* = 81) of the first experiment, whereas the three panels in B reflect the three‐way interaction of the reduced data set (removing P_2_ values of 0 and 1; *N* = 52)

When considering the relative difference in sperm length between brothers across all *N* = 81 competitive mating trials with complete data (including both mating orders in the paired assay), there was some evidence for a competitive advantage of longer sperm as estimated by the proportional paternity of the second male (P_2_ = 0.63 [0.55–0.71]). In the absence of a direct main effect of the relative difference in sperm lengths (*β* = 0.27 ± 0.32, $\chi_{1}^{2}$ = 0.67, *p* = 0.47), there was a weak, albeit statistically non‐significant trend for P_2_ being jointly explained by the relative difference in sperm lengths interacting with the relative difference in copula durations (*β* = 0.74 ± 0.37, $\chi_{1}^{2}$ = 4.04, *p* = 0.07; Figure 1a). P_2_ increased with the relative difference in copula durations (positive [main] effect: *β* = 1.06 ± 0.32, $\chi_{1}^{2}$ = 11.00, *p* = 0.004), as well as with the relative difference in male body sizes (positive [main] effect: *β* = 1.10 ± 0.33, $\chi_{1}^{2}$ = 10.45, *p* = 0.006). However, there was no effect of female body size (*β* = 0.36 ± 0.33, $\chi_{1}^{2}$ = 1.21, *p* = 0.32) or the type of assay (paired versus unpaired: $\chi_{1}^{2}$ = 3.05, *p* = 0.11).

Because P_2_ was either 0 or 1 for 29 of the 81 trials, which could (but need not) indicate (unsuccessful) copulations without sperm transfer by the second or the first male, respectively, we repeated the above analysis for those 52 trials with at least some paternity by both competitors (strong inference subset of data). P_2_ was still biased toward the second male (P_2_ = 0.56 [0.48–0.63]), although its 95% confidence interval now included equal paternity between competitors. Among its predictors, the interaction between the relative differences in sperm lengths and copula durations shifted from negative to positive with increasing female body size, as indicated by a significant three‐way interaction between these three variables (*β* = 0.79 ± 0.29, $\chi_{1}^{2}$ = 7.40, *p* = 0.02; Figure 1b). The relative difference in sperm lengths also positively affected P_2_ (*β* = 0.65 ± 0.19, $\chi_{1}^{2}$ = 10.58, *p* = 0.008), and there was a weak, statistically non‐significant interactive effect with female body size (*β* = 0.41 ± 0.20, $\chi_{1}^{2}$ = 4.38, *p* = 0.07). All other main or interactive effects of these three variables were unimportant (all $\chi_{1}^{2}$ ≤ 0.76, *p* ≥ 0.38). P_2_ was further explained by the relative difference in male body sizes (*β* = 0.60 ± 0.18, $\chi_{1}^{2}$ = 10.63, *p* = 0.008) and was, on average, weakly higher in the unpaired compared to paired assay (*β* = 1.05 ± 0.20, $\chi_{1}^{2}$ = 5.15, *p* = 0.04).

### 3.2 Tests using phenoloxidase selection lines

**FIGURE 2:**
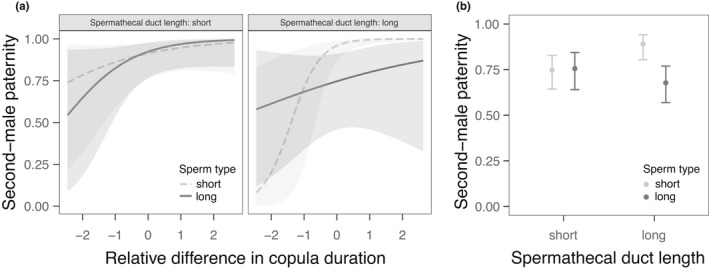
(a) The predicted proportion of offspring sired by the second male P_2_ (with 95% confidence bands) increases with the relative difference in copula durations between the competitors, more strongly for short‐spermed males in longer female spermathecal ducts (lines resulting from phenoloxidase (PO) selection; second experiment, *N* = 117 trials). (b) Mean P_2_ (± 95% CIs) of long‐ versus short‐spermed males in females with short or long ducts (reduced data set with P_2_ values of 0 and 1 removed; *N* = 64)

The experiment using selection lines revealed a three‐way interaction between female and second‐male selection regime and relative copulation durations between males (*N* = 117 trials across 24 combinations of male and female replicate selection lines; *β* = −2.36 ± 1.09, $\chi_{1}^{2}$ = 4.97, *p* = 0.04; Figure 2a). Hence, when competitive fertilization occurred in females with short (or normal) spermathecal ducts (high PO), second males with short sperm (high PO regime) sired most offspring, regardless of the relative copulation durations between competitors. Second males of the long‐sperm (low PO) regime, however, lost paternity if their copulation was not longer than that of their competitor. When females had long spermathecal ducts, it was the short‐spermed second males that lost paternity in case of relatively short copulations, although they clearly outperformed their long‐spermed competitors whenever they copulated for longer. Besides this three‐way interaction, however, the remaining interactions, and all main effects, were not statistically significant (all $\chi_{1}^{2}$ ≤ 3.05, *p* ≥ 0.16).

When excluding the cases with P_2_ of 0 or 1 (as above), leaving *N* = 64 trials, the non‐significant three‐way interaction and the two‐way interactions involving copula duration did not improve model fit and were therefore removed from the model ($\chi_{1}^{2}$ < 1.43, *p* > 0.23). Among the remaining predictors, a two‐way interaction between male and female PO types indicated a short‐sperm advantage in females with long spermathecal ducts (*β* = −1.40 ± 0.55, $\chi_{1}^{2}$ = 6.39, *p* = 0.02), but equal paternity success of sperm types when spermathecal ducts were short (Figure 2b). Additionally, there was a positive effect of the relative difference in copula durations (*β* = 0.41 ± 0.13, $\chi_{1}^{2}$ = 9.45, *p* = 0.007), and a negative effect of female body size (*β* = −1.67 ± 0.81, $\chi_{1}^{2}$ = 1.06, *p* = 0.05). The relative difference in male body sizes or sex‐specific PO types did not affect P_2_ (all $\chi_{1}^{2}$ ≤ 1.06, *p* ≥ 0.33).

## 4 DISCUSSION

Our major finding was that longer sperm did not confer any consistent paternity advantage in yellow dung flies, the classic model species for studies of sperm competition and sexual selection (Simmons et al., 2020). In all tests, paternity was primarily biased toward the second of two competing males, consistent with numerous previous reports for this species (reviewed in Simmons, 2001; Simmons et al., 2020). In tests taking advantage of temperature‐mediated sperm length variation across 81 brother–brother competitions to minimize genetic influences, this second‐male advantage was greater when second males had relatively longer sperm and copulated for longer (Figure 1). In the reduced, stronger inference data set (excluding P_2_ = 0 or 1), the interaction between male traits was further influenced by the size of the female, and thus likely by the length of their spermathecal ducts (Parker et al., 1999; Schwarzenbach, 2006; Thüler et al., 2011). An interaction between female spermathecal duct length and both relative sperm length and relative copula duration of competitors was also observed in our second experiment, using flies from PO selection lines with (genetically) correlated responses in sperm length and female spermathecal duct length (Schwarzenbach, 2006; Schwarzenbach & Ward, 2006, 2007). Taken together, these results point toward complex but subtle interactions between multiple female and male reproductive traits that likely underlie competitive fertilization success, rather than any straightforward expected advantage of longer sperm due to, for instance, swimming speed or sperm displacement capacity (Fitzpatrick & Lüpold, 2014; Lüpold & Pitnick, 2018; Simmons & Fitzpatrick, 2012).

Our experimental manipulation to generate brothers of different sperm length was prompted by previous, somewhat surprising results that warmer rearing temperature systematically elongates sperm of emerging male yellow dung flies, for unknown functional reasons (Blanckenhorn & Hellriegel, 2002). Hot temperatures are also well known to affect various other aspects of reproductive success of this (and many other) species, including juvenile mortality or reproductive behaviour, and likely also including male fertility (Blanckenhorn et al., 2014, 2021). It is therefore possible that our hot temperature treatment may have induced physiological effects on sperm, ultimately affecting male fertility beyond the morphological changes in focus here (Sales et al., 2019; Walsh et al., 2019). In the extreme, it is possible that larger sperm are more competitive (e.g. at swimming), but other thermally induced defects offset this advantage and so confounded our results in the experiment competing brothers. Blanckenhorn et al. (2014) assessed in detail the effects of hot temperatures on yellow dung fly life history, including whether copula duration, female oviposition, and subsequent survivorship of offspring is compromised by hot temperatures. Male reproductive success, expectedly at least in part reflecting sperm fertility, was indeed lower at hotter temperatures, but this was equally the case for female success/fertility. Although this experiment did not directly assess fertilization success of *sperm* as a function of temperature, we conclude that the long‐known reduction of yellow dung fly survivorship at hot temperatures is not strongly affected by physiological effects on sperm independent of their size (figure 6 in Blanckenhorn et al., 2014, 2021).

Of the traits contributing to differential paternity outcomes in the yellow dung fly, copula duration is the best studied and has previously often been shown to covary positively with the number of sperm transferred and, ultimately, paternity share (Bussière et al., 2010; Demont et al., 2021; Parker & Simmons, 1994; Simmons et al., 1999). Prolonged copulations therefore likely indeed confer a simple numerical advantage among sperm, consistent with the general raffle principle predicted by classical sperm competition theory (reviewed in Parker & Pizzari, 2010). Whether sperm of different males are mixed or displaced and ultimately discarded (as is the case in the yellow dung fly: Parker et al., 2010), relative sperm numbers are likely to contribute to competitive fertilization success, with increased sperm production or transfer being a near‐ubiquitous response to female multiple mating among the species studied so far (Birkhead & Møller, 1998; Lüpold, de Boer, et al., 2020; Parker, 1982; Parker & Pizzari, 2010; Simmons & Fitzpatrick, 2012). Our results therefore are broadly in line with this general pattern.

Beyond copula duration predicting relative sperm numbers, their interactions with relative sperm lengths in both our experiments nevertheless indicate that competitive fertilization may not merely result from numerical advantages (Parker, 1990, 1993). Yet, our two experiments were inconsistent with regard to the direction of these interactive effects on P_2_, and partly influenced by the size of the females and/or their spermathecal ducts. In our first experiment competing brothers, longer sperm tended to enhance the effect of relative copula duration (and putative sperm numbers), according to our reduced (strong inference) data set particularly when competing in relatively large females (with larger sperm‐storage organs; Thüler et al., 2011; Figure 1). Competitive advantages of relatively longer sperm have been reported in various species (Fitzpatrick & Lüpold, 2014; Pitnick, Hosken, & Birkhead, 2009; Simmons & Fitzpatrick, 2012). Selection for longer sperm is thought to be particularly prevalent in small, internally fertilizing organisms, including insects in which the highly confined space of the female reproductive tract causes direct sperm interactions and often displacement of resident sperm from female sperm‐storage structures by incoming sperm (Immler et al., 2011; Parker et al., 2010). Although the precise mechanism remains unknown, at least in *Drosophila* it has been shown in different experimental contexts that longer sperm are better at displacing shorter rival sperm, or at resisting displacement by them (Lüpold et al., 2012; Manier et al., 2013; Miller & Pitnick, 2002). Genetic correlations between sperm length, sperm displacement ability, and the size of female sperm‐storage organs (Lüpold et al., 2016; Miller & Pitnick, 2002) may thus promote selection for longer sperm to explain the taxonomically widespread examples of interspecific covariation between these traits (reviewed by Lüpold & Pitnick, 2018).

Despite the subtle long‐sperm effect found in the brother–brother competitions, our other test uncovered no such effect, as also documented in several other taxa (Boschetto et al., 2011; Dziminski et al., 2009; Gage & Morrow, 2003; García‐González & Simmons, 2007; Laskemoen et al., 2010; Morrow & Gage, 2001; Simmons et al., 2003). In our second experiment using artificially selected yellow dung flies, a three‐way interaction between female spermathecal duct length, relative sperm lengths and copula durations revealed that second males with *shorter* sperm achieved higher P_2_, however only when competing in females with long spermathecal ducts (Figure 2). An advantage of shorter sperm in longer ducts is difficult to explain, unless small sperm size translates into more sperm transferred to females. Such a conclusion seems plausible given the corresponding copula durations, but is complicated by the realized *positive* genetic correlation between sperm and spermathecal duct lengths following selection on PO in yellow dung flies (Schwarzenbach, 2006; see also Hosken et al., 2001; Thüler et al., 2011). This correlated response particularly contrasts with another experimental evolution study in which direct manipulation of the mating system, and thus the degree of post‐mating sexual selection, resulted in no divergence in sperm or spermathecal size, but instead in significantly larger testes and relatively higher paternity shares in polyandrous yellow dung fly lines (Hosken et al., 2001). It is therefore likely that the paternity advantage of second males with shorter sperm (from high PO lines) in females with longer spermathecal ducts (from low PO lines) was caused by some other correlated effects of immunocompetence, rather than sperm length per se (also see Arnaud et al., 2005). Since female yellow dung flies typically have three spermathecae (but occasionally four) and store sperm differentially between them (Bussière et al., 2010; Demont et al., 2021; Walters et al., 2022), it is also possible, albeit speculative, that females derived from different genetic and selective background varied in how they stored and used sperm due to different spermathecal duct lengths. Such effects could further extend to female accessory gland secretions known to affect sperm survival (Thüler et al., 2021), either by differential fluid composition or sperm sensitivity. Even if the functional links between these findings remain elusive, they do point toward the female × male × male interactions underlying paternity found here being sensitive to environmental variation, and to the limits of generalizing experimental findings on fitness outcomes.

By using copula duration instead of directly quantifying sperm numbers, our interpretations involving sperm numbers necessarily remain tentative despite validation of this proxy in many previous studies (cited above). Based on paternity results alone we were also unable to distinguish different scenarios explaining the many cases of single‐male paternity suggesting either complete sperm displacement or lack of sperm transfer (i.e. possible male infertility, e.g. induced by high temperature). By either including or excluding these cases, we necessarily erred on one or the other extreme of what might have been the true fitness outcomes. Further, the precise timing of second copulations relative to oviposition and the number of eggs laid between copulations (if any) were not recorded, thus missing some other potentially subtle effects on paternity outcomes by female differential sperm use or male sperm allocation in response to female oviposition (Demont et al., 2021). Although our results confirm prior studies (see above) in suggesting that such confounding effects are likely limited, some caution is still warranted in their interpretation. Nonetheless, to the extent that they are biologically relevant, the often subtle interactions reported here highlight the non‐independence of various ejaculate traits potentially contributing to competitive fertilization success beyond mere sperm morphology (e.g. seminal fluid, sperm motility, viability or displacement ability; Fitzpatrick & Lüpold, 2014; Lüpold & Pitnick, 2018; Simmons & Fitzpatrick, 2012), and likely female influences on sperm performance and biases between competing sperm via variation in their selective environment (Eberhard, 1996; Firman et al., 2017; Gasparini et al., 2020; Pitnick, Wolfner, & Suarez, 2009; including yellow dung flies: Demont et al., 2021; Thüler et al., 2021). To date, idiosyncratic fitness outcomes among females and competing males have been studied primarily at the genotypic level (reviewed in Lüpold, Reil, et al., 2020) and have been thought to weaken sexual selection on male traits by reducing the contribution of their residual variance to paternity (Birkhead, 1998; Neff & Pitcher, 2005; Pitnick & Brown, 2000). However, as sex‐specific fitness traits and the interactions between them are typically context‐ or condition‐dependent, this might generate enough variation to facilitate directional sexual selection and even the evolution of extreme phenotypes, while at the same time overcoming the depletion of genetic variation in the population through such selection (Lüpold, Reil, et al., 2020).

## AUTHOR CONTRIBUTIONS

**Ane Timenes Laugen:** Conceptualization (supporting); formal analysis (equal); investigation (lead); methodology (equal); validation (equal); visualization (equal); writing – original draft (supporting); writing – review and editing (supporting). **Paquita Hoeck:** Data curation (supporting); formal analysis (supporting); investigation (supporting); methodology (supporting); validation (supporting); writing – original draft (supporting). **Wolf Blanckenhorn** Conceptualization–Lead, Data curation–Lead, Formal analysis–Supporting, Funding acquisition–Lead, Methodology–Supporting, Project administration–Lead, Resources–Lead, Supervision–Lead, Writing – original draft–Lead, Writing – review and editing–Equal. **David Hosken:** Conceptualization (supporting); formal analysis (supporting); funding acquisition (supporting); investigation (supporting); methodology (supporting); resources (supporting); supervision (supporting); validation (supporting); visualization (supporting); writing – original draft (supporting); writing – review and editing (supporting). **Klaus Reinhold:** Funding acquisition (supporting); investigation (supporting); methodology (supporting); validation (supporting); writing – original draft (supporting). **Luc Bussière:** Conceptualization (supporting); data curation (supporting); formal analysis (equal); investigation (supporting); methodology (supporting); project administration (supporting); software (supporting); supervision (supporting); validation (supporting); visualization (supporting); writing – original draft (supporting); writing – review and editing (supporting). **Gioia Schwarzenbach:** Data curation (supporting); formal analysis (supporting); investigation (equal); methodology (supporting); project administration (supporting); validation (supporting); writing – original draft (supporting). **Stefan Lüpold:** Conceptualization (supporting); data curation (equal); formal analysis (lead); methodology (supporting); project administration (supporting); software (lead); validation (equal); visualization (lead); writing – original draft (lead); writing – review and editing (lead).

## CONFLICT OF INTEREST

The authors have no conflicts of interest to declare.

### PEER REVIEW

The peer review history for this article is available at https://publons.com/publon/10.1111/jeb.14073.

## Data Availability

All data and code are deposited on the Figshare Data Repository, doi: https://doi.org/10.6084/m9.figshare.20184029.
